# Ipecac in Uterine Hæmorrhages

**Published:** 1892-06

**Authors:** J. R. Haynes

**Affiliations:** Indianapolis, Indiana; Clinical Bureau, I. H. A.


### IPECAC IN UTERINE HÆMORRHAGES.

J. R. Haynes, M. D., Indianapolis, Indiana.

Clinical Bureau, I. H. A.

Was called to see Mrs. T., aged twenty-two years, light complexion, brown hair, blue eyes, rather small in stature, would weigh about one hundred pounds, married, and the mother of one child about two and a half years old. As near as I could learn, she had had a miscarriage about one year before, and had a poor recovery from it; was treated by a "regular" and thoroughly dosed, or such was the report.

She had been feeling well, had made no complaints, was sitting up with some light sewing in hand, when she was taken suddenly with a severe uterine hæmorrhage; she was placed upon the bed and I was sent for, with the request to come as soon as it was possible.

When I arrived, she had fainted two or three times. I found her pulseless, face pale, and so much exsanguinated that she could not speak, so that all of the information that I could get must be obtained from some of the rest of the family, and that was but very little.

The hæmorrhage had run through her clothing, through the bed, and a large pool had collected upon the floor.

She was flowing very rapidly; a large stream was gushing from the uterus, so that there was no time to wait. Whatever was done must be done at once, or death would take place in a few moments.

The flow was of a bright red (purely arterial), the lower limbs were bathed in a cold perspiration; hands were cold and damp; the abdomen felt hot yet damp with perspiration; the flow would come in large gushes, and life was ebbing out very rapidly.

The color of the discharge was of a bright red, and did not coagulate easily, but lay upon the floor in a liquid pool.

I considered that all of the symptoms that I could get pointed to Ipecac. A small dose of Ipecac$^{10m}$ was placed in a half-glass of water and one teaspoonful was given as soon as possible.

It acted like magic, for in less than one minute there was a change for the better. It was repeated in fifteen minutes, when the active hæmorrhage ceased. I waited for an hour to see if there would be any return (which there was not) when I left Sac-lac. in water to be given one teaspoonful every hour, and left with the promise that if any alarming symptoms should make their appearance that I should be notified at once. I would not allow even her wet bloody clothing to be changed, but to slip some dry clothes under her next to the skin to make her as comfortable as possible. There was a slight oozing of the discharge for two days when it entirely ceased.

She was very weak and prostrated after the tremendous flow; and for these conditions she was given at intervals three doses of China$^{10m}$; she made a good getting up, and in a week was able to be up and dressed, and in two weeks was able to come down-town.

Mrs. L——, aged thirty-one, light complexion, brown hair, blue eyes, medium height, would weigh about one hundred and ten pounds, quite active when in health, lively disposition, full of fun, fond of a good joke (especially when upon others) was taken suddenly with a severe uterine hæmorrhage, which came away in gushes very fluid, and of a bright red color. It did not coagulate easily; looked like fresh arterial blood. An oppressed heavy feeling over the lower abdomen; heavy ache through the small of the back; throbbing sensation through the head, partial illusions before the eyes upon motion, a fainty nausea, which seemed to come from the stomach; tongue coated white, rather thirsty; quite gloomy, thought that she would bleed to death; somewhat restless (I thought from fright), hands and feet covered with a cold perspiration. Had been told by some old women that she was likely to bleed to death, which had a very depressing effect upon her; felt rigors if the clothing was moved or she stirred; had been well up to the time of this sudden attack, and could not assign any reason for it. Here was another picture of Ipecac. A small dose was placed in water and one teaspoonful was given and told to continue it every half-hour until four doses were taken should the activity of the flow continue; but as soon as there were any symptoms of an improvement to stop it. A powder of Sac-lac. was left to take its place. The violent hæmorrhage ceased in about an hour, and by the next morning, the discharge had entirely ceased; and there was no further trouble.

Mrs. K——, aged twenty-eight; light complexion, brown hair, blue eyes, tall and slim; very active; must be in motion; at times very gloomy, and from no apparent cause; would weigh about one hundred pounds; married, and the mother of one child, which is seven years old, and has never been pregnant since the first time. Was taken suddenly with active uterine hæmorrhage, which was of a bright red color, and for such a midget was very active; would coagulate when cold; had the smell of fresh blood; she had a heavy ache in the lower abdomen, the skin over the abdomen felt hot, and a slight hot perspiration. Had to urinate often, in small quantities. The flow would come on with gushes; felt faint and nauseated; throbbing headache; worse through the forehead; face pale and bloodless, which I attributed to the loss of blood; a sort of sallow look; tongue coated white; some thirst, dead, heavy soreness in the throat; mouth clammy; some cough, with sticky mucus in the larynx; felt sore through the whole chest; no appetite; considerable flatulence in the bowels; heavy, aching pain in the uterine region; hands and feet cold and clammy; somewhat restless; the flow was worse upon motion, yet was restless, and could not bear to keep quiet; very gloomy, and thought that she was not going to get well again, and what would become of her little girl; that she would never get up again, so that she could attend to her, which was the most that she cared for.

Ipecac$^{10m}$ in water, one teaspoonful every hour, and as soon as the flow began to cease, to throw it away, and take a powder of Sac-lac., prepared in the same way. After the third dose the hæmorrhage was so much less that she did not continue it longer, but had the Sac-lac. prepared, and took that, and by the next morning the hæmorrhage had diminished to a slight discharge, which continued for about two days longer, and then ceased entirely.

She has had no further trouble in that way now for many months, or I should have heard from her, as I see her often.

Mrs. B——, aged twenty-four; dark complexion, black hair and eyes; rather chubby built; would weigh about one hundred and ten pounds; married; one child, a little girl, about two years old; of a rather gloomy disposition, going fully half-way to meet trouble. Was taken suddenly with uterine hæmorrhage, which was of a bright red color, which came away in gushes, which commenced with a fainty nausea, with some retching; face bloodless; pulse small and quick, one hundred and twenty; feet cold and clammy; abdomen hot; a clammy perspiration on the face; a sickening headache; aching over the whole head; heavy ache through the small of the back; aggravated by motion; sore aching through the front of the chest; spasmodic spells of coughing, which aggravated the hæmorrhage, which would come in gushes; a stuffed-up feeling in the head (probably from crying); heavy pressure through the lower abdomen; and before one of the gushes would have a considerable griping in the uterine region; gloomy and despondent; knew that she would bleed to death; thought that she felt the best when she kept perfectly quiet, but could not remain so; flow aggravated by her moving, which would also cause a gush to pass off, and that would make her more gloomy and restless.

Ipecac$^{10m}$ in water, one teaspoonful every hour until four doses had been taken, or, as soon as the hæmorrhage seemed to get low, to throw it away, and take a powder of Sac-lac. prepared in the same way. At the fourth dose the active hæmorrhage ceased, and the Sac-lac. was prepared and taken; the next day there was a slight discharge, which grew gradually less, and did not entirely cease until the third day; and so far there has been no further trouble in that line.

There is a large number of remedies that have a bright red discharge from the uterus; but so far as I know, none of them have the peculiar characteristics of Ipecac. It seems to stand out very prominently in all of its characteristics, and cannot be easily mistaken for any other remedy. One very peculiar characteristic is that the flow in active hæmorrhage is the peculiar gushing, which could be compared to that of a pump when the handle is vigorously worked; the stream does not cease, but at every pulsation of the heart there is a peculiar gush, which is not credited to any other remedy, so far as I am aware; and then the blood does not easily coagulate, but remains fluid for sometime, especially when active uterine hæmorrhage takes place.

